# HHV-6 and EBV reactivation in relapsing remitting multiple sclerosis: Disability, progression, and inflammation links

**DOI:** 10.1016/j.isci.2025.113048

**Published:** 2025-07-05

**Authors:** Abbas F. Almulla, Aristo Vojdani, Yingqian Zhang, Elroy Vojdani, Michael Maes

**Affiliations:** 1 Sichuan Provincial Center for Mental Health, Sichuan Provincial People’s Hospital, School of Medicine, University of Electronic Science and Technology of China, Chengdu 610072, China; 2 Key Laboratory of Psychosomatic Medicine, Chinese Academy of Medical Sciences, Chengdu 610072, China; 3 Department of Psychiatry, Faculty of Medicine, Chulalongkorn University, and King Chulalongkorn Memorial Hospital, the Thai Red Cross Society, Bangkok, Thailand; 4 Medical Laboratory Technology Department, College of Medical Technology, The Islamic University, Najaf, Iraq; 5 Immunosciences Lab, Inc., Los Angeles, CA 90035, USA; 6 Cyrex Laboratories, LLC, Phoenix, AZ 85034, USA; 7 Regenera Medical, Los Angeles, CA 90025, USA; 8 Department of Psychiatry, Medical University of Plovdiv, Plovdiv, Bulgaria; 9 Research Center, Medical University of Plovdiv, Plovdiv, Bulgaria; 10 Research and Innovation Program for the Development of MU - PLOVDIV– (SRIPD-MUP), Creation of a Network of Research Higher Schools, National Plan for Recovery and Sustainability, European Union – NextGenerationEU, Plovdiv, Bulgaria; 11 Kyung Hee University, 26 Kyungheedae-ro, Dongdaemun-gu, Seoul 02447, Korea

**Keywords:** Microbiology, Virology

## Abstract

Reactivation of human herpesvirus 6 (HHV-6) and Epstein-Barr virus (EBV) is observed in multiple sclerosis (MS). This study investigates immunoglobulins (Ig)G, IgM, and IgA responses to EBV nuclear antigen EBNA (peptide 386–405), HHV-6 (peptide 300–322) and EBV (peptide 243–268) deoxyuridine-triphosphatase (dUTPase), and different immune profiles in 58 patients with relapsing remitting MS (RRMS) compared to 60 healthy controls. IgA/IgG/IgM were measured utilizing enzyme-linked immunosorbent assays, and cytokines, chemokines, and growth factors utilizing multiplex immunoassays. RRMS patients showed significantly increased IgG, IgA, and IgM responses to all three viral antigens. IgG and IgM to HHV-6 dUTPase discriminated RRMS patients from controls with 91.5% accuracy. Neural network analysis combining EBV-dUTPase antibodies and immune profiles yielded 97.1% predictive accuracy. IgG/IgM responses to dUTPases correlated with Expanded Disability Status Scale/MS Severity Score and aberrations in M1 macrophage, Th17 profiles, and overall immune activation. HHV-6 and EBV reactivation contribute to RRMS through cytokine-driven immune activation.

## Highlights

• RRMS is characterized by elevated IgG, IgA, and IgM targeting HHV-6 and EBV antigens
• IgM to HHV-6/EBV dUTPase and IgG to EBV-dUTPase strongly differentiate RRMS from controls
• IgG/IgM to HHV-6 and EBV dUTPase links with MS severity, disability, and immune imbalance

## Graphical abstract

**Figure:**
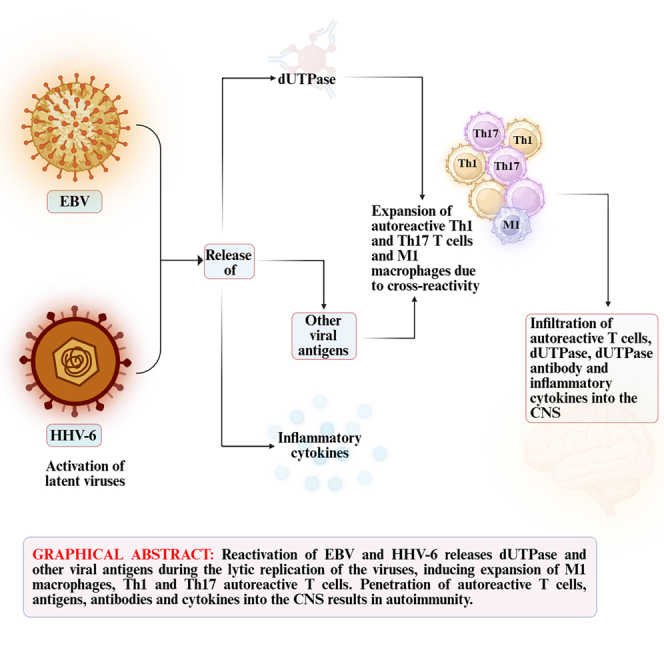

## Introduction

Multiple sclerosis (MS) is a chronic, autoimmune-mediated disease that primarily affects the central nervous system (CNS). MS is defined by the demyelination, neuroinflammation, and neurodegeneration of the nervous system,^1^^,^^2^^,^^3^ resulting in a wide range of symptoms, including impaired motor function, and sensory abnormalities.^4^^,^^5^^,^^6^ The global burden of MS is substantial, with approximately 2.8 million people affected worldwide, exhibiting significant geographical variability in prevalence.^7^^,^^8^ The manifestation of MS varies greatly, encompassing a wide range of severity levels and diverse clinical trajectories, such as relapsing-remitting MS (RRMS), primary-progressive MS, and secondary-progressive MS.^9^

Recent research highlights the critical role of immune-inflammatory responses and oxidative stress in the onset of MS.^10^^,^^11^^,^^12^ Activation of immune-inflammatory response system (IRS) pathways in individuals with MS are demonstrated by elevated concentrations of proinflammatory cytokines in both the blood and cerebrospinal fluid (CSF).^13^ Even remitted RRMS patients show indicants of activation of the (a) IRS with M1 macrophage, T helper 1 (Th1), T helper 17 (Th17) activation, (b) compensatory immune response system (CIRS) with Th2 and T regulatory (Treg) cell activation, and (c) chemokines and growth factor (GF) networks.^5^ Additionally, some studies found an association between the severity of clinical disabilities, as measured by the Expanded Disability Status Scale (EDSS) and indicators of oxidative and nitrosative stress.^11^^,^^14^

Latent viral infections, particularly by Epstein-Barr virus (EBV) and human herpesvirus 6 (HHV-6), have been increasingly implicated in the pathogenesis of MS.^15^ Some studies have detected EBV and HHV-6 in the CNS and CSF of MS patients, with evidence suggesting a link to disease activity.^16^^,^^17^^,^^18^ However, other studies have found no significant differences in viral detection between MS and non-MS patients.^19^^,^^20^^,^^21^ These inconsistencies highlight the need for further research with larger cohorts to clarify the role of these viruses in MS. The immunoglobulin (Ig) response to these viral proteins has been proposed as a marker for MS onset and progression.^22^^,^^23^ For example, elevated levels of IgM directed to HHV-6 (IgM-HHV-6) and IgG-EBV antibodies have been observed in early-stage MS patients.^24^ EBV nuclear antigen 1 (EBNA-1) immunoglobulin G (IgG) levels are associated with the disabilities observed in MS (as assessed with the EDSS) and with gadolinium-enhancing lesions.^25^

Some studies found associations between EBNA1 IgG titers and indicators of disease severity, including EDSS scores and MRI lesion activity.^25^^,^^26^^,^^27^ Other studies, however, have not supported these findings, suggesting that while EBNA1 seropositivity is recognized as a risk factor for the onset of MS, its impact on disease progression remains ambiguous.^28^^,^^29^ In addition, the associations between IgM/IgA/IgG levels directed to HHV-6 and EBV deoxyuridine 5′-triphosphate nucleotidohydrolase (dUTPase) were not investigated in MS. The herpesvirus dUTPases are a reliable indicator of viral activity, as they are only expressed during viral replication and reactivation or active “abortive” infection.^30^^,^^31^ Their expression indicates active viral involvement, differentiating true viral reactivation from latent persistence. Additionally, as a pathogen-associated molecular pattern (PAMP), herpes dUTPases engage Toll-like receptors (TLRs) and activate nuclear factor kappa B (NF-κB), resulting in induction of proinflammatory cytokines.^32^^,^^33^ These immunomodulatory properties suggest a potential role in sustaining chronic inflammation in RRMS.

Thus, this study examined whether (a) the remitted phase of RRMS is accompanied by signs of EBV and HHV-6 replication or reactivation as assessed with IgG/IgA/IgM responses to EBV-and HHV-6-dUTPase and EBV nuclear antigen (EBNA) peptide 386–405); and (b) whether the EBV or HHV-6 reactivation indices are associated with disabilities and progression of disease and the immune profiles observed in RRMS. This study investigated emerging serological markers of viral replication, specifically the dUTPases of HHV-6 (peptide 300–322) and EBV (peptide 243–268), as alternatives to the conventional focus on EBNA1 and viral DNA. Those data are analyzed using deep learning methods and will be integrated with immunological profiles, including IRS and CIRS.

## Results

### Socio-demographic and clinical characteristics of MS

Table 1 presents the sociodemographic data, DOI, and scores from various disability assessments, including ADLs, the EDSS, and the MSSS, comparing RRMS patients with healthy controls. There were no significant differences observed in age, sex, marital status, or BMI between the groups. Significant differences were found between RRMS patients and healthy controls in employment status, and smoking status. RRMS patients demonstrated significantly elevated scores on ADL, EDSS, and MSSS scores.

**Table 1:** Demographic and clinical data in patients with relapsing remitting multiple sclerosis (RRMS) and healthy controls (HC)

Variables	HC (*n* = 63)	RRMS (*n* = 55)	F/X^2^	df	*p* value
Age (years)	32.3 (7.1)	29.8 (8.4)	3.48	1/124	0.064
Sex (M/F)	33/30	37/17	2.699	1	0.133
Married/Single (Ma/S)	26/37	31/24	2.679	1	0.139
Employment (No/Yes)	2/28	46/9	46.784	1	<0.001
Smoking Yes/No	44/19	50/5	8.044	1	0.006
BMI	26.3 (4.5)	24.4 (3.5)	4.11	1/124	0.052
ADLs	14 (0)	13.22 (1.19)	MWU	–	<0.001
DOI	0(0)	5.58 (4.94)	MWU	–	<0.001
EDSS	0(0)	1.027(0.114)	MWU	–	<0.001
MSSS	0(0)	1.71(1.206)	MWU	–	<0.001

M, male; F, female; Ma, married; S, single; BMI, body mass index; ADLs, activity of daily living; DOI, duration of illness; EDSS, Expanded Disability Status Scale; MSSS, Multiple Sclerosis Severity Score.

### Differences in the immune responses among the study groups

Table 2 shows that RRMS patients demonstrated higher IgG, IgA, and IgM responses to EBNA 386–405, EBV-dUTPase, and HHV-6-dUTPase as compared to healthy controls. In addition, M1 macrophage, Th17, chemokines, GF, IRS, CIRS, and IRS+CIRS profiles were significantly increased in MS patients.

**Table 2:** Differences in immunoglobulin (Ig) IgG/IgA/IgM reactivity against Epstein-Barr virus (EBV) and human herpesvirus type 6 (HHV-6) proteins and immune profiles between patients with relapsing remitting multiple sclerosis (RRMS) versus healthy controls (HC)

Variables	HCs (*n* = 63)	RRMS (*n* = 55)	F/X^2^	df	*p*
IgG-EBNA (peptide 386–405)	0.619(0.115)	1.528(0.124)	26.720	4/113	<0.0001
IgM-EBNA (386–405)	0.298(0.029)	0.580 (0.031)	42.120	4/113	<0.0001
IgA-EBNA (386–405)	0.290 (0.026)	0.527(0.028)	35.210	4/113	<0.0001
IgG-EBV-dUTPase (243–268)	0.836(0.098)	1.927(0.105)	53.964	4/113	<0.0001
IgM-EBV-dUTPase (243–268)	0.609(0.061)	1.234(0.066)	44.831	4/113	<0.0001
IgA-EBV-dUTPase (243–268)	0.511(0.076)	1.367(0.081)	55.131	4/113	<0.0001
IgG-HHV-6-dUTPase (300–322)	0.307(0.036)	0.739(0.038)	62.764	4/113	<0.0001
IgM-HHV-6-dUTPase (300–322)	0.326(0.032)	0.694(0.035)	55.459	4/113	<0.0001
IgA-HHV-6-dUTPase (300–322)	0.312(0.032)	0.628(0.034)	43.104	4/113	<0.0001
M1 macrophage (z scores)	−0.573(0.112)	0.573(0.112)	8.161	4/105	0.005
Th1 (z scores)	−0.280(0.134)	0.280(0.134)	67.938	4/105	<0.0001
Th17 (z scores)	−0.652(0.108)	0.652(0.108)	53.600	4/105	<0.0001
Chemokines (z scores)	−0.607 (0.113)	0.607(0.113)	61.870	4/105	<0.0001
GFs (z scores)	−0.630(0.109)	0.630(0.109)	58.254	4/105	<0.0001
IRS (z scores)	−0.621(0.111)	0.621(0.111)	152.375	4/105	<0.0001
CIRS (z scores)	−0.790(0.087)	0.790(0.087)	152.438	4/105	<0.0001
IRS+CIRS (z scores)	−0.793(0.088)	0.793(0.088)	8.161	4/105	0.005

Ig, Immunoglobulin; EBNA, EBV nuclear antigen; dUTPase, deoxyuridine-triphosphatase; HHV-6, human herpesvirus 6; EBV, Epstein-Barr virus; M1, macrophage M1; Th, T helper; GFs, growth factors; IRS, immune response system; CIRS, compensatory immune response system; IRS+CIRS, combined index of IRS and CIRS activation.

In the restricted group of RRMS patients, there were no significant point-biserial correlations between the use of natalizumab and any of the viral data, except IgG-dUTPAse (r = −0.300, *p* = 0.026, *n* = 55, without false discovery rate p correction for multiple testing). The use of beta-interferon-1β was significantly associated (without p-correction) with lowered levels of IgM-EBNA (r = −0.283, *p* = 0.036), IgA-EBNA (r = −0.336, *p* = 0.012), IgM-EBV-dUTPase (r = −0.305, *p* = 0.023), and IgA-HHV-6-dUTPase (r = −0.267, *p* = 0.049). However, after using FDR p correction, all significance disappeared. The results show that the findings of the study were not affected by the drug state of the patients.

Binary logistic regression was used to identify the most important indicators for RRMS. In the logistic regression model, the control group functioned as the reference group and RRMS as the dependent variable. The IgA, IgG, and IgM responses to EBNA-386-405, EBV-dUTPase, and HHV-6-dUTPase, as well as age, sex, BMI, and smoking, were the independent factors in this investigation.

Table 3**,** model #1 indicates that IgG- and IgM-HHV-6-dUTPase were significantly and positively associated with RRMS with an effect size 0.803 and an overall accuracy of 91.5% (sensitivity = 87.3%, and specificity = 95.2%). Model #2 shows that these results did not change after including age, sex, BMI, and smoking.

**Table 3:** Results of binary logistic regression analysis with relapsing remitting multiple sclerosis (RRMS) as dependent variable

Dichotomies	Explanatory Variables	B	SE	Wald	*p*	OR	95% CI
**RRMS patients versus healthy controls**							

Model #1	IgG-HHV-6-dUTPase	4.622	1.043	19.621	<0.001	101.68	13.15; 785.93
	IgM-HHV-6-dUTPase	1.697	0.547	9.630	0.002	5.45	1.86; 15.93
Model #2	IgG-HHV-6-dUTPase	4.415	1.056	17.473	<0.001	82.69	10.43; 655.48
	IgM-HHV-6-dUTPase	1.734	0.572	9.178	0.002	5.66	1.84; 17.37

Ig, Immunoglobulin; DUTPase, deoxyuridine-triphosphatase; HHV-6, human herpesvirus 6; BMI, body mass index.

Model #1: Nagelkerke R square: 0.803; sensitivity: 95.2%; specificity: 87.3%; overall accuracy 91.5%.

Model #2: this model includes age, sex, BMI, and smoking (forced entry); these 4 variables were non-significant in the regression, and the impact of the HHV-6 markers on RRMS did not change after forced entry of these possible confounders.

**Figure 1:**
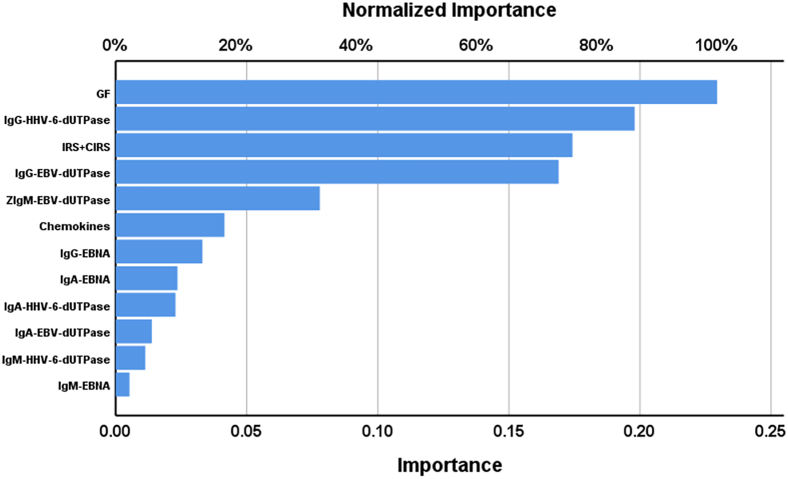
Results of neural network analysis demonstrating the importance chart The output variables are the diagnosis of relapsing remitting multiple sclerosis and healthy controls. Input variables are growth factors (GF), immunoglobulin (Ig)G- human herpes virus 6 (HHV-6) deoxyuridine-triphosphatase (dUTPase) (peptide 300–322), IRS+CIRS (immune activation index), IgG-Epstein-Barr virus (EBV) dUTPase (peptide 243–268), IgM-EBV-dUTPase, chemokines, IgG-EBV-nuclear antigen (EBNA) (peptide 386–405), IgA-EBNA, IgA-HHV6-dUTPase, IgA-EBV-dUTPase, IgM-HHV-6-dUTPase, IgM-EBNA.

Table 4 shows the results of neural network analyses. NN#1 revealed the characteristics of a first neural network model that differentiates RRMS patients from healthy controls. This model was constructed using 2 hidden layers with 2 units in hidden layer 1, 2 in hidden layer 2, and 2 units in the output layer. The error term was significantly lower in the testing than in the training sample, while the percentage of incorrect classifications was similar between the three samples, indicating that the model was not overtrained. This solution was better than the logistic regression, with a predictive accuracy (computed in the holdout sample) of 97.7% (sensitivity = 100% and specificity = 96%) and an area ROC curve of 0.997. The topmost important biomarkers (Figure 1) were in descending order of importance: the GF profile, IgG-HHV-6-dUTPase, IRS+CIRS, and IgG- and IgM-EBV-dUTPases.

**Table 4:** Results of neural network analysis with relapsing remitting multiple sclerosis (RRMS) as output variable

Components	Models	NN#1	NN#2
Input Layer	Number of units	12	4
Hidden layers	Number of hidden layers	2	2
	Activation function	Hyperbolic tangent	Hyperbolic tangent
	Number of units in hidden layer 1	2	3
	Number of units in hidden layer 2	2	2
Output layer	Dependent variable	RRMS Patients vs. controls	RRMS Patients vs. controls
	Number of units	2	2
	Activation function	Identity	Identity
Training	Error term	1.426	2.602
	% incorrect classifications	2.3%	1.9%
	Sensitivity, specificity	100%, 95%	100%, 96.9%
Testing	Sum of Squares error	1.032	0.861
	% incorrect classifications	4.2%	0.0%
	Sensitivity, specificity	100%, 90%	100%, 100%
	Area under the ROC curve	0.997	1
Holdout	% incorrect classifications	19.4%	2.9%
	Predictive accuracy: Sensitivity, specificity	100%, 96%	95.5%, 100%

ROC, receiver operating characteristics.

**Figure 2:**
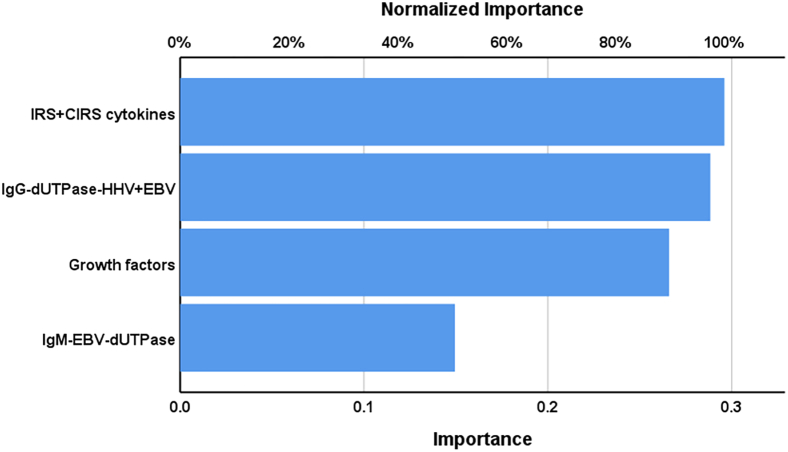
Results of neural network analysis demonstrating the importance chart The output variables are the diagnosis of relapsing remitting multiple sclerosis and healthy controls. Input variables are IRS+CIRS (an immune activation index), a z unit composite score of IgG-EBV-dUTPase and IgG-HHV-6-dUTPase, growth factors, and IgM-EBV-dUTPase.

We have rerun the above neural network analysis using the top 5 most important variables combined with a z unit composite score of IgG-EBV-dUTPase and IgG-HHV-6-dUTPase and detected that using 4 of those input variables yielded a comparable differentiation of RRMS from controls. The features of this second neural network model (NN#2) are displayed in Table 4. This model was constructed using 2 hidden layers with 3 nodes in hidden layer 1, 2 in hidden layer 2, and 2 units in the output layer. The error term was significantly lower in the testing than in the training sample, while the percentage of incorrect classifications was quite similar between the training, testing, and holdout samples. The predictive accuracy of this neural network model (computed in the holdout sample) was 97.1% (sensitivity = 95.5% and specificity = 100%) with an area under the ROC curve of 1. The topmost important biomarkers (Figure 2) were in descending order of importance: IRS+CIRS, the composite of IgGs directed against dUTPases, growth factors, and IgM-EBV-dUTPase.

### Immune responses to reactivated latent viruses predict the severity of RRMS

**Figure 3:**
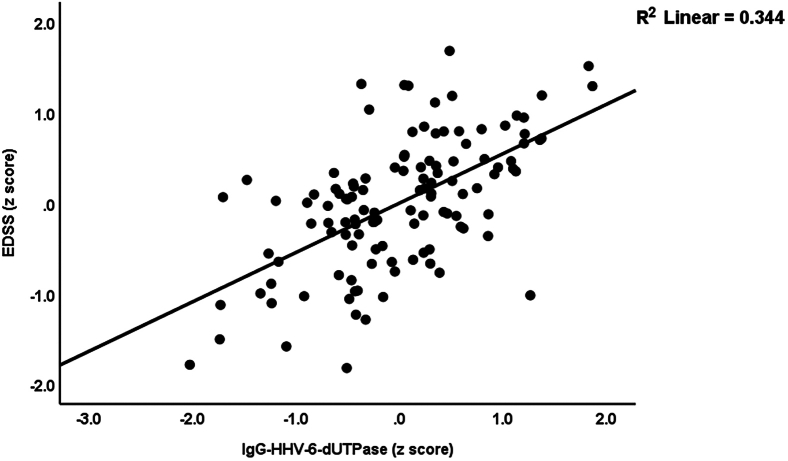
Partial regression of the Expanded Disability Status Scale (EDSS) on immunoglobulin (Ig)G directed against human herpes virus-6 (HHV-6) deoxyuridine-triphosphatase (dUTPase), *p* < 0.001

Table 5 presents the results of multiple regression analyses with the EDSS and MSSS scores as dependent variables. The independent variables include the IgG/IgM/IgA levels against the three viral antigens. First, we compute the effects of these viral data on disabilities (allowing for the effects of age, sex, smoking, and BMI). Consequently, we enter immune profiles into multiple regression analyses. Regression analysis #1 shows that 65.4% of the variance in EDSS scores can be explained by IgG and IgM against HHV-6-dUTPase, and age. Figure 3 illustrates the partial regression of the EDSS score on IgG against HHV-6-dUTPase. Regression analysis #2 shows that the model accounts for 77.6% of the variance in the EDSS score; IRS+CIRS, IgG, and IgM against HHV-6-dUTPase, age, and sex were the significant explanatory variables. In regression analysis #3, IgG-HHV-6-dUTPase, IgM-EBV-dUTPase, and age predict 52.1% of the variance in MSSS scores. Regression #4 reveals that 64.4% of the variance in the MSSS score was explained by the regression on IgG-HHV-6-dUTPase, IRS+CIRS, IgA-HHV-6-dUTPase, and age.

**Table 5:** Results of multiple regression analysis with the Expanded Disability Status Scale (EDSS) and Multiple Sclerosis Severity Score (MSSS) as dependent variables

Dependent variables	Explanatory variables	Coefficients of input variables			Model statistics			
		β	t	*p*	R^2^	F	df	*p*
#1. EDSS	Model				0.654	71.98	3/114	<0.001
	IgG-HHV-6-dUTPase	0.547	7.73	<0.001				
	IgM-HHV-6-dUTPase	0.295	4.21	<0.001				
	Age	−0.142	−2.51	0.013				
#2. EDSS	Model				0.776	72.20	5/104	<0.001
	IRS+CIRS	0.448	7.45	<0.001				
	IgG-HHV-6-dUTPase	0.351	5.50	<0.001				
	IgM-HHV-6-dUTPase	0.182	3.05	0.003				
	Age	−0.118	−2.45	0.016				
	Sex	−0.111	−2.39	0.019				
#3. MSSS	Model				0.521	22.56	3/114	<0.001
	IgG-HHV-6-dUTPase	0.497	6.62	<0.001				
	IgM-EBV-dUTPase	0.306	4.13	<0.001				
	Age	−0.142	−2.28	0.024				
#4. MSSS	Model				0.646	47.85	4/105	<0.001
	IgG-HHV-6-dUTPase	0.268	3.10	0.003				
	IRS+CIRS	0.388	5.33	<0.001				
	IgA-HHV-6-dUTPase	0.252	3.31	0.001				
	Age	−0.130	−2.16	0.033				
#5 EDSS (in patients)	Model				0.083	4.82	1/53	0.033
	IgM-EBV-dUTPase	0.289	2.20	0.033				
#6 MSSS (in patients)	Model				0.205	13.64	1/53	0.001
	IgA-HHV-6-DUTPase	0.452	3.693	0.001				
#7 IgA-IgM dUTPase (in patients)	Model				0.185	5.914	2/52	0.005
	DOI	−0.354	−2.739	0.008				
	Age	0.347	2.687	0.010				

Ig, Immunoglobulin; dUTPase, deoxyuridine-triphosphatase; HHV-6, Human herpesvirus 6; EBV, Epstein-Barr virus; DOI, duration of illness; IRS, immune response system; CIRS, compensatory immunoregulatory system; IgA+IgM dUTPase, sum of z scores of IgM and IgA to EBV and HHV-6-dUTPases.

We conducted additional regression analyses (#5 and #6), which were performed in the restricted study group of RRMS patients. Regression analysis #5 shows that 8.3% of the variance in EDSS score was explained by the regression on IgM-EBV-dUTPase (positively associated with the outcome). IgA-HHV-6-dUTPase accounted for 20.5% of the variance in the MSSS score (regression #6). Lastly, we also examined whether the IgA/IgM values to the dUTPases were predicted by DOI. Toward this end we computed a z composite score using the IgA/IgM responses to EBV and HHV-6-dUTPases. We found that this composite score was inversely associated with DOI and positively with age. Both variables together explained 18.5% of the variance of the composite score.

### Reactivated latent viruses and immune profiles

**Figure 4:**
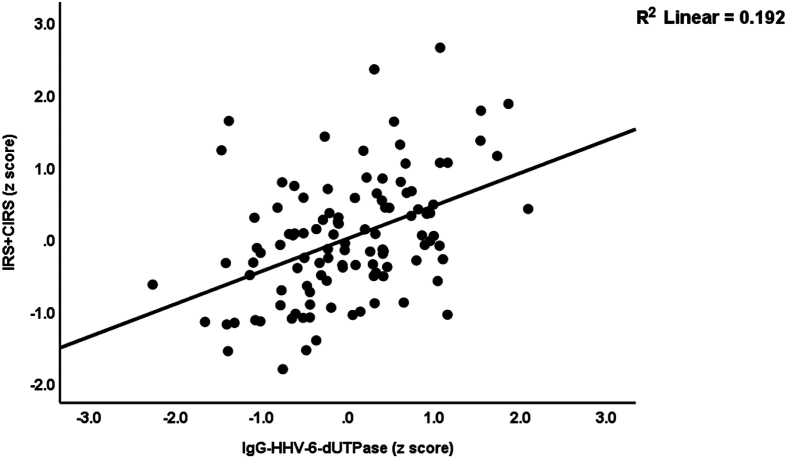
Partial regression of the immune activation index (IRS+CIRS) on immunoglobulin (Ig)G directed against human herpes virus-6 (HHV-6) deoxyuridine-triphosphatase (dUTPase), *p* < 0.001

Table 6 shows the associations between immune profiles and the EBV and HHV-6 data. The latter were entered as explanatory variables, whereas the immune profiles were entered as dependent variables. In addition, we allowed for the effects of age, sex, BMI, and smoking. Regression #1 shows that the model accounts for 17.3% of the variance in the M1 macrophage profile, with IgG-HHV-6-dUTPase and IgM-EBV-dUTPase emerging as significant predictors. Regression #2 shows that these two variables explained 27.2% of the variance in the Th17 profile. Th1 was not predicted by any of the variables. Regression #3 demonstrates that 21.8% of the variance in GFs is significantly explained by IgG-HHV-6-dUTPase and IgM-EBV-dUTPase, both of which are positively associated with the Th17 axis. In regression #4, 16.3% of the variance in IRS is significantly explained by IgG-HHV-6-dUTPase. Regression #5 shows that 52.3% of the variance in CIRS is significantly explained by IgG-HHV-6-dUTPase, IgM-EBNA, and smoking. Regression #6 reveals that 40.7% of the variance in the IRS+CIRS ratio is explained by IgG-HHV-6-dUTPase and IgM-EBV-dUTPase. Figure 4 exhibits the partial regression of the IRS+CIRS on IgG against HHV-6-dUTPase. Regression #7 indicates that IgG-HHV-6-dUTPase explains 15.8% of the variance in the chemokine profile.

**Table 6:** Results of multiple regression with the immune profiles as dependent variables

Dependent variables	Explanatory variables	Coefficients of input variables			Model statistics			
		β	t	*p*	R^2^	F	df	*p*
#1. M1 macrophage	Model				0.173	11.22	2/107	<0.001
	IgG-HHV-6-dUTPase	0.258	2.458	0.016				
	IgM-EBV-dUTPase	0.215	2.051	0.043				
#2. Th17	Model				0.272	19.94	2/107	<0.001
	IgG-HHV-6-dUTPase	0.380	3.858	<0.001				
	IgM-EBV-dUTPase	0.205	2.082	0.040				
#3. GFs	Model				0.218	14.88	2/107	<0.001
	IgG-HHV-6-dUTPase	0.296	2.903	0.004				
	IgM-EBV-dUTPase	0.233	2.284	0.024				
#4. IRS	Model				0.163	21.04	1/108	<0.001
	IgG-HHV-6-dUTPase	0.404	4.587	<0.001				
#5 CIRS	Model				0.523	38.76	3/106	<0.001
	IgG-HHV-6-dUTPase	0.432	5.166	<0.001				
	IgM-EBNA-386-405	0.303	3.778	<0.001				
	Smoking Y/N	−0.163	−2.252	0.026				
#6 IRS+CIRS	Model				0.407	36.74	2/107	<0.001
	IgG-HHV-6-dUTPase	0.448	5.039	<0.001				
	IgM-EBV-dUTPase	0.272	3.056	0.003				
#7 Chemokines	Model				0.158	20.29	1/108	<0.001
	IgG-HHV-6-dUTPase	0.398	4.505	<0.001				

Ig, Immunoglobulin; dUTPase, deoxyuridine-triphosphatase; HHV-6, human herpesvirus type 6; EBV, Epstein-Barr virus. M1, macrophage M1; Th, T helper; GFs, growth factors; IRS, Immune response system; CIRS, compensatory immunoregulatory system; IRS+CIRS, combined index of IRS and CIRS activation.

## Discussion

### Indications of EBV and HHV-6 reactivation in RRMS

The first major finding of this study is that remitted RRMS patients demonstrated significantly increased levels of IgG/IgA/IgM to HHV-6 and EBV viral proteins, including EBNA-386-405, EBV-dUTPase-243-268, and HHV-6-dUTPase-300-322 as compared to normal controls.

The detection of antibody responses to HHV-6 in the CSF, or the presence of HHV-6 DNA in serum, CSF supernatant, or CSF leukocytes, is typically uncommon during clinical remission of MS, highlighting the limitations of these markers for a definitive diagnosis.^34^ Nevertheless, the measurement of IgG to dUTPase (and by inference IgM and IgA to the dUTPase), are more specific markers of ongoing viral replication. Elevated IgG levels against herpesvirus dUTPases suggest ongoing viral replication through an abortive lytic cycle, where viral proteins are produced without forming new virions.^35^^,^^36^ It should be stressed that this kind of replication markers does not necessarily indicate increased viral load.^35^

The current study extends previous evidence which reported elevated IgG responses to EBV EBNA-1 and HHV-6 in MS patients.^22^^,^^23^ Our results extend those of previous studies which reported increased antibodies (IgM and IgG) to HHV-6 and EBV.^16^^,^^24^^,^^37^ A recent meta-analysis reported an association between MS and EBV and HHV-6.^15^ In addition, previous studies have suggested an association between HHV-6 and MS based on immunological and molecular data, indicating that a subset of MS patients may show HHV-6 reactivation.^38^^,^^39^^,^^40^

Previous research on MS consistently indicates elevated IgG antibodies against EBNA1, whereas IgM is infrequently observed outside of acute EBV infection or reactivation. In this regard, EBNA1 exhibits shared pentapeptide sequences with CNS-related proteins, such as GlialCAM, MBP, MOG, MAG, and SYN1.^41^^,^^42^ This process may induce B cell activation, resulting in IgM production even without active viral replication. Moreover, a dysregulated humoral response in MS may involve an abnormal activation of memory B cells secreting IgM antibodies.^43^^,^^44^

### Indications of EBV and HHV-6 reactivation and severity of illness

Importantly, we also observed that a combination of IgG, IgM, and IgA responses to HHV-6 and EBV-dUTPase significantly predicted the severity of disabilities due to MS (as measured by the EDSS) and disease progression (as assessed with the MSSS scale). These findings extend previous research that linked IgG against HHV-6 with an increased risk of MS progression, including relapses and disability.^37^^,^^45^ Lundström and Gustafsson reported that HHV-6A infection may play a role in the early stages of MS.^18^ Anti-HHV-6A/B IgG titers also hold the potential to predict impending relapses in MS disease.^46^ The presence of HHV-6 DNA in serum samples of MS patients coupled with increased IgG response to HHV-6 suggests a possible association between virus reactivation and disease progression.^39^ However, other studies contradict these results by reporting that the parameters of EBV and HHV-6 reactivation were not always associated with disability or progression of MS disease.^47^^,^^48^

### Pathways linking EBV and HHV-6 reactivation with RRMS

Both increased EBV and HHV-6 replication may, through different mechanisms, induce MS relapses. HHV-6 reactivation may trigger or aggravate autoimmunity and tissue damage associated with MS lesion development, possibly through molecular mimicry or excessive complement activation.^38^ Lanz et al. reported a high-affinity molecular mimicry between the EBNA1 and the CNS protein GlialCAM in MS patients.^41^ Clonally expanded B cells in the CSF of MS patients produce antibodies that cross-react with EBNA1 and GlialCAM, facilitated by post-translational modifications. This cross-reactivity was shown to exacerbate disease in a mouse model of MS, providing a mechanistic link between EBV infection and MS pathobiology.^41^ Serum levels of the neurofilament light chain, a biomarker of neuroaxonal degeneration, increased following EBV seroconversion, indicating a temporal relationship between EBV infection and the onset of MS.

Interestingly, we found that the IgA responses to EBV and HHV-6 dUTPases were inversely correlated with the duration of illness. These findings may suggest that the replication ability of these viruses decreases with illness duration. Such an effect could play a role in the age-associated decreases in annualized relapse rate.^49^

### Viral replication and the drug state of the RRMS patients

We found that the use of disease-modifying therapies (DMTs) did not impact the results of the present study. Indeed, the drug treatments had no significant impact on the results or resulted in a very small effect size of around 3% of the variance. Nevertheless, we found a trend toward a suppressant effect of beta-interferon-1β on the dUTPase values. Beta-interferon-1β treatment has been reported to decrease HHV-6 viral load during MS relapses, though this effect is not observed during remission.^50^ Treatment with natalizumab is associated with the reactivation of several latent viruses, including HHV-6^51^ and EBV.^52^^,^^53^ This reactivation is believed to result from impaired immune surveillance, a consequence of natalizumab’s impact on T cell migration and phenotype.^51^^,^^54^ Moreover, the measurement of anti-HHV-6 IgG titers has been proposed as a potential biomarker for the clinical response to DMTs.^46^ High IgG response to EBNA-1 correlated with MS disease activity on MRI and disability progression along with clinical response to natalizumab.^25^^,^^55^

### EBV and HHV-6 antibodies and immune profiles

Almulla et al. (2023) demonstrated that elevated levels of Th-1 and Th-17 cytokines, are significant predictors of disabilities in MS patients.^5^ This finding is particularly relevant given that the upregulation of these immune profiles may contribute to autoimmune processes through diverse mechanisms.^56^ Most importantly, the current report shows that the EBV and HHV-6 replication indices are significantly correlated with activated immune profiles, including overall immune functions (IRS+CIRS), M1, Th-17, IRS, CIRS, and GF immune profiles. For example, around 20% of the variance in the M1 and Th-17 profiles was explained by the combined effects of IgM or IgG directed to EBV-dUTPase and HHV-6-dUTPase. While 16.3% of the variance in the IRS profile was associated with IgG HHV-6-dUTPase, 40% of the variance in CIRS profile was associated with both EBV (IgM) and HHV-6 (IgG)-dUTPases. Thus, it appears that dUTPases of both EBV and HHV-6 may drive IRS and CIRS activation.

dUTPases are recognized as PAMPs that may exacerbate immune pathology in various diseases including MS.^33^ These viral proteins could influence disease pathophysiology by modulating the host innate immune system specifically by activating the TLR-4 complex and NF-κB, thereby triggering cytokine production (M1, Th-1, and Treg cytokines).^57^^,^^58^^,^^59^

In addition, EBV-dUTPase contributes to latency by increasing the production of IL-21 and activin A.^36^ HHV-6 seropositivity was linked to altered pro-inflammatory cytokine levels in MS patients.^60^ The correlation between HHV-6 reactivation and serum IL-12 concentrations during disease activity suggests that HHV-6 reactivation is linked to MS aggravation.^61^

Furthermore, the current study provides evidence that combining EBV and HHV-6 replication indices with GF and IRS+CIRS immune profiles yields a predictive accuracy of 97.1% in diagnosing RRMS. Thus, EBV and HHV-6 replication and its consequences such immune activation, may be used to externally validate the diagnosis of RRMS. Previous research has focused on other biomarkers, such as neurofilament light chain, chitinase 3-like proteins, and microRNAs.^62^^,^^63^ The combination of several CSF and plasma proteins, such as IL-12B, CD5, MIP-1a, and CXCL9, had a diagnostic efficacy similar to traditional markers, including IgG index and neurofilament light chain.^64^ In addition, serum biomarkers, including adhesion molecules and matrix metalloproteinase-9, may help monitor disease activity.^65^

In conclusion, our results indicate that even in the remitted phase of RRMS and despite treatments with DMTs, HHV-6, and EBV replication may be present and, consequently, may contribute to new relapses. IgG/IgA/IgM responses to EBV and HHV-6 dUTPase combined with immune profiles allow to externally validate the diagnosis of RRMS. EBV and HHV-6 replication are associated with disabilities and progression of the disease. Our results suggest that the pathophysiology of RRMS involves both the reactivation/replication of latent viruses and the activation of immune-inflammatory pathways. Different mechanisms related to EBV and HHV-6 replication may explain the onset and relapsing of MS, including TLR-4 activation, inflammation, expansion of M1 macrophages and Th-17 cells, molecular mimicry, infiltration of autoreactive T cells and antibodies, neuroinflammation, and breakdown of myelin sheaths. Our findings highlight that EBV and HHV-6 dUTPases contribute to these pathways and indicate that EBV/HHV-6 dUTPases are important drug targets to treat RRMS and prevent future relapses.

### Limitations of the study

When interpreting the results of this study, certain limitations must be acknowledged. Considering the role of oxidative and nitrosative stress markers in the pathophysiology and progression of MS,^66^^,^^67^ it would be beneficial to examine these biomarkers in conjunction with indicators of latent viral reactivation. Also, upon their availability, monoclonal antibodies made against EBV and HHV-6 dUTPases should be applied to each dUTPase and human tissue antigen in order to determine whether or not antibodies detected in the sera of MS patients in this study are specific or cross-reactive. The cross-sectional design of this study does not allow to make inferences on causal relationships between viral reactivation biomarkers and severity of RRMS. Consequently, longitudinal studies are essential to determine the temporal relationship between viral reactivation and disease progression. The findings from our Iraqi cohort may not generalize worldwide due to genetic and environmental variations. Nevertheless, the findings from the Iraqi cohort in this study deserve replication in other countries and cultures. Although the accuracy of our neural network model was very high (accuracy in a holdout sample of 97.1%), future research should employ a larger sample size to validate the predictive accuracy of the biomarker tool developed in the current study.

## Resource availability

### Lead contact

For additional information and resource requests, please contact the lead contact, Professor Dr. Michael Maes (dr.michaelmaes@hotmail.com).

### Materials availability

This research did not yield any novel reagents or materials. The STAR Methods section offers detailed information about all kits and reagents employed in this study.

### Data and code availability

The SPSS file produced and/or examined in this study can be obtained from the corresponding author (MM) upon reasonable request. This study did not involve the development or use of any custom code or algorithms.

## Acknowledgments

The authors extend their sincere appreciation to the Neuroscience Center of Alsader Medical City in Al-Najaf province, Iraq, for their crucial assistance in gathering the data. Funding for the project was provided by the C2F program at Chulalongkorn University in Thailand, grant no 64.310/436/2565 to A.F.A., the Thailand Science Research, and Innovation Fund at Chulalongkorn University (HEA663000016), and a Sompoch Endowment Fund (Faculty of Medicine) MDCU (RA66/016) to M.M. For performance of all antibody assays, funds were provided by Immunosciences Lab., Inc., Los Angeles, CA, USA, and Cyrex Labs, LLC, Phoenix, AZ, USA.

## Author contributions

A.F.A. managed the blood sample collection and patient-related procedures. A.V. and A.F.A. conducted the serum biomarker quantification. M.M. performed the statistical analysis. M.M., A.V., and E.V. performed visualization. The initial draft was written by A.F.A. and M.M., and subsequently revised by A.V., Y.Z., and E.V. All authors approved of the last version.

## Declaration of interests

The authors declare no conflicts of interest.

## STAR★Methods

### Key resources table

**Table:** 

REAGENT or RESOURCE	SOURCE	IDENTIFIER
**Antibodies**		

IgA-EBNA-386-405	Biosynthesis (Lewisville, TX, USA)	NA
IgG-EBNA-386-405	Biosynthesis (Lewisville, TX, USA)	NA
IgM-EBNA-386-405	Biosynthesis (Lewisville, TX, USA)	NA
IgA- HHV-6-duTPase	Biosynthesis (Lewisville, TX, USA)	NA
IgG- HHV-6-duTPase	Biosynthesis (Lewisville, TX, USA)	NA
IgM- HHV-6-duTPase	Biosynthesis (Lewisville, TX, USA)	NA
IgA- EBV-DUTPase	Biosynthesis (Lewisville, TX, USA)	NA
IgG- EBV-DUTPase	Biosynthesis (Lewisville, TX, USA)	NA
IgM- EBV-DUTPase	Biosynthesis (Lewisville, TX, USA)	NA

**Biological samples**		

Serum	Neuroscience Center of Alsader Medical City in Al-Najaf province, Iraq	NA

**Chemicals, peptides, and recombinant proteins**		

Carbonate buffer	Sigma Aldrich	https://www.sigmaaldrich.com/IQ/en/product/sigma/c3041?srsltid=AfmBOoo3kK8yFVQFYtueRGc4MFfYHWNxqEhO4BtO3h0kk2Lqzvy6ZkGS
Bovine Serum Albumin	Sigma Aldrich	https://www.sigmaaldrich.com/IQ/en/substance/bovineserumalbumin123459048468

**Critical commercial assays**		

Bio-Plex Pro™ Human Cytokine 27-plex Assay	Bio-Rad	https://www.selectscience.net/product/bio-plex-pro-tm-human-cytokine-27-plex-assay/#description

**Software and algorithms**		

SPSS	IBM	RRID:SCR_016479

### Experimental model and study participant details

#### Participants

Between September 2021 and March 2022, 58 RRMS patients from the Neuroscience Center of Alsader Medical City in Al-Najaf province, Iraq, were enrolled in this case-control study. They were in the remitted phase of RRMS. The diagnosis of MS was confirmed using the McDonald criteria^68^ by a senior neurologist. Additionally, sixty healthy individuals from the same demographic area, including hospital staff, their acquaintances, and medical workers were recruited. Controls were matched for age and sex and region of residence to the patients.

All participants were screened to exclude those with current Axis-I neuropsychiatric disorders, such as schizophrenia, bipolar disorder, psycho-organic disorders, and substance use disorders (except nicotine dependence). The study also excluded individuals with medical conditions like diabetes mellitus, cardiovascular diseases, thyroid disorders, renal or liver diseases, gastrointestinal conditions, oncologic disorders, other (auto)immune, and neuroinflammatory and neurodegenerative diseases, including psoriasis, COPD, inflammatory bowel disease, Parkinson’s disease, and Alzheimer’s disease.

Written informed consent was obtained from all RRMS patients or their parents/legal guardians, as well as from control participants prior to their inclusion in the study. This research received ethical approval from the institutional ethics board of the College of Medical Technology at The Islamic University of Najaf, Iraq (document number 11/2021). Adherence to ethical standards was ensured by following both Iraqi and international guidelines, including the World Medical Association Declaration of Helsinki, The Belmont Report, CIOMS guidelines, and the International Conference on Harmonization of Good Clinical Practice (ICH-GCP). Our Institutional Review Board (IRB) complies with the International Guidelines for Human Research Safety.

#### Ethical approval and consent to participate

The investigation received approval from the Ethics Committee of the College of Medical Technology at the Islamic University of Najaf, Iraq (Document No. 11/2021). All procedures adhered to both Iraqi and international ethical standards and written informed consent was obtained from all patients and control participants.

### Method details

#### Clinical assessment

A senior neurologist conducted a semi-structured interview to gather sociodemographic and clinical data, including duration of illness (DOI). The same neurologist utilized the EDSS^69^ to evaluate clinical disability, and the MSSS^70^ to assess the progression of disability over time. Activities of daily living (ADL) were measured using the Arabic-translated index of ADL.^71^ Body mass index (BMI) was determined by dividing the participant’s weight in kilograms by their height in meters squared.

#### Biomarkers assays

Blood samples were collected from fasting participants between 7:30 and 9:00 a.m. using venipuncture with disposable syringes. The collected blood was allowed to clot at room temperature for 15 min before being centrifuged at 3500 RPM for 10 min. The resulting serum was aliquoted into Eppendorf tubes for use in various assays. An enzyme-linked immunosorbent assay (ELISA) was employed to detect antibodies (IgA, IgG, IgM) specific to HHV-6-duTPase, EBV-DUTPase, and EBNA-386-405 peptides, which were synthesized from Biosynthesis (Lewisville, TX, USA). The methodology described in previous studies,^72^^,^^73^ in brief, 100 microliters of different peptides at a concentration of 5 micrograms per mL in 0.1 M carbonate buffer ph 9.5 were added to different wells of ELISA plates. After incubation, washing and blocking with 2% BSA, 100 microliters each of the control and MS patients’ sera at a dilution of 1:50 for IgA and 1:100 for IgG and IgM determination were added to duplicate wells. Plates were then incubated, washed, and secondary antibodies were then added to each plate. Finally, after repeated washing and the addition of substrate, the color development was measured, and indices were calculated using different sera as calibrators and controls. As controls, we also used several wells coated with bovine serum albumin, human serum albumin, and beef extracts followed by the addition of all other reagents. The ODs of these control wells were similar to the blank wells.

Our following findings support the specificity of antibodies detected against EBV and HHV-6 dUTPases.

(1) In the ELISA assay we coated several wells with bovine serum albumin, human serum albumin and beef extract. The ELISA ODs obtained did not differ from the background or blank wells.
(2) We compared the indices of IgG, IgA and IgM antibody obtained against EBV EBNA-386-405, EBV dUTPase-243-268 and HHV-6 dUTPase-300-322 in a plot, and did not find simultaneous elevation against two or all three antigens simultaneously. This means that sera which reacted strongly with EBV EBNA did not react with EBV dUTPase and/or HHV-6 dUTPase. Similarly, sera which reacted with EBV-dUTPase did not react with HHV-6 dUTPase simultaneously, and vise-versa.
(3) Based on pioneering studies by Williams, Cox and Ariza,^33^ we did not find significant amino acid sequence similarities between EBV and HHV-6 dUTPases. Furthermore, we did not find any amino acid overlap between EBV dUTPase peptide 243–268 and HHV-6A/6B dUTPase peptide 300–322. Although all of this shows no cross-reactivity between the EBV dUTPase peptide 243–268 and HHV-6 dUTPase peptide 300322 used in this study, the final answer will be given only upon the availability of monoclonal or polyclonal antibodies made against these dUTPases and reacting them against each other as well as with various tissue antigens.

Additionally, a comprehensive assessment of cytokines/chemokines/growth factors, relevant to M1 macrophage activation, Th-1, Th-17, IRS, CIRS, IRS+CIRS, chemokine, and growth factor profiles was performed using the Bio-Plex Pro™ Human Chemokine Assays from Bio-Rad Laboratories, Inc. (Hercules, USA) as described previously.^5^ Table S1, shows the cytokines/chemokines/growth factors assessed in this study. As described previously,^74^^,^^75^ our analysis utilized immunofluorescence (IF) values of cytokines/chemokines/growth factors and used the blank subtracted IF values to compute z unit-based composite scores reflecting different immune profiles.^5^^,^^74^^,^^76^^,^^77^ Table S2 lists the immune profiles assessed in the current report.

### Quantification and statistical analysis

We employed IBM’s SPSS 29 for all statistical analyses in this study. Analysis of variance (ANOVA) was used to compare continuous variables between study groups, while contingency table analysis was used to examine the association between categorical variables. Pearson’s product moment and point-biserial correlations were used to examine associations among scale variables and between scale and binary data, respectively. Multiple comparisons and associations were p-corrected for False-Discovery Rate (FDR). To explore the relationships between IgA, IgG, and IgM responses and RRMS diagnosis, we conducted a binary logistic regression analysis, using RRMS as dependent variable and healthy controls as the reference group. This analysis accounted for potential confounding factors such as age, gender, smoking, and BMI. Key results included estimates of effect sizes using Nagelkerke pseudo-R square, Wald statistics with *p*-values, odds ratios with 95% confidence intervals (CI), and unstandardized regression coefficient B and standard error (SE) of B, with the Wald statistic equaling the ratio of the B coefficient to its SE, squared. For predicting outcomes related to the EDSS and the MSSS in RRMS patients, we utilized multivariate regression analysis, adjusting for demographic variables such as age, gender, and BMI. Manual and stepwise methods (including automatic linear modeling with overfit prevention) were applied, the latter with entry and exit criteria set at *p*-values of 0.05 and 0.06, respectively. This analysis provided model metrics, including standardized beta coefficients, degrees of freedom (df), *p*-values, R^2^, and F-statistics. To examine heteroskedasticity, we used the White test and the modified Breusch-Pagan test, and we assessed collinearity by evaluating tolerance and the variance inflation factor. A two-tailed design was used for all tests, with a significant threshold of 0.05.

In addition, we developed multilayer perceptron neural network models to distinguish RRMS patients from healthy controls. These models included IgG/IgM/IgA responses to EBNA-386-405, EBV-DUTPase, HHV-6-DUTPase with or without immune profiles as input variables. The neural networks featured a feedforward architecture with two hidden layers, each potentially containing up to eight nodes, and they were trained in batch-type sessions for a maximum of 250 epochs. Training stopped when further reductions in the error term were not observed. The analysis comprises three samples, namely training, testing and holdout samples. The latter estimates the predictive value of the model. The significance and relative importance of the input variables were visualized using an importance chart, and metrics such as error, relative error, and misclassification rates were calculated by comparing predicted versus actual values.

An *a priori* power analysis using G∗Power 3.1.9.7 determined that a minimum sample size of 90 individuals was needed to detect differences in an ANCOVA (analysis of covariance) test, assuming a power of 0.8, a significance level (p) of 0.05, two groups, and 4 covariates and an effect size of 0.3.
