# Supplementary material for: HHV-6 and EBV reactivation in relapsing remitting multiple sclerosis: Disability, progression, and inflammation links
Source: iScience. 2025 Jul 5;28(8):113048. doi: 10.1016/j.isci.2025.113048 (PMC12309957; doi:10.1016/j.isci.2025.113048)
Supplement: Document S1. Tables S1 and S2 [file mmc1.pdf]

iScience, Volume *28*

Supplemental information

HHV-6 and EBV reactivation in relapsing remitting multiple sclerosis: Disability, progression, and inflammation links

Abbas F. Almulla, Aristo Vojdani, Yingqian Zhang, Elroy Vojdani, and Michael Maes

Supplemental information

Table S1. Cytokine, chemokines and growth factors examined in the current study

**Protein abbreviations**	Gene Symbol	**Protein name**
IL-1β	IL1B	Interleukin-1β
IL-1RA	IL1RN	Interleukin-1 receptor antagonist
IL-2	IL2	Interleukin-2
IL-4	IL4	Interleukin-4
IL-5	IL5	Interleukin-5
IL-6	IL6	Interleukin-6
IL-7	IL7	Interleukin-7
CXCL8	CXCL8	C-X-C motif chemokine ligand 8 (IL-8)
IL-9	IL9	Interleukin-9
IL-10	IL10	Interleukin-10
IL-12p70	IL12	Interleukin-12
IL-13	IL13	Interleukin-13
IL-15	IL15	Interleukin-15
IL-17	IL17	Interleukin-17
CCL11	CCL11	Eotaxin
FGF2	FGF2	Fibroblast growth factor 2, Basic fibroblast growth factor
G-CSF	CSF3	Granulocyte Colony Stimulating Factor, Colony Stimulating Factor 3 (Granulocyte)
GM-CSF	CSF2	Granulocyte-macrophage colony-stimulating factor, Colony-stimulating factor 2
IFN-γ	IFNG	*Interferon-γ*
CXCL10	CXCL10	C-X-C motif chemokine ligand 8, Interferon gamma-induced protein 10 (IP10)
CCL2	CCL2	C-C Motif Chemokine Ligand 2 (MCP1)
CCL3 / MIP-1α	CCL3	Macrophage inflammatory protein-1 alpha, C-C Motif Chemokine Ligand 3
PDGF	PDGFA	Platelet Derived Growth Factor Subunit A
CCL4 / MIP-1β	CCL4	C-C Motif Chemokine Ligand 4, Macrophage Inflammatory Protein 1-Beta, Lymphocyte Activation Gene 1 Protein

CCL5 /RANTES	CCL5	C-C Motif Chemokine Ligand 5, Regulated Upon Activation, Normally T-Expressed, And Presumably Secreted
TNF-α	TNF	Tumor Necrosis Factor-Alpha
VEGF	VEGFA	Vascular Endothelial Growth Factor

Table S2. Description of the immune profiles used in this study

Immune Profile	Members
**M1 macrophage**	IL-1β, sIL-1RA, IL-6, TNF-α, CXCL8, CCL3
**T helper-1**	IL-2, IFN-γ, IL-12
**T helper-17**	IL-6, IL-17
**IRS**	IL-1β, IL-6, TNF-α, CXCL8, CCL3, IL-2, IFN-γ, IL-12, IL-17, IL-15, G-CSF, GM-CSF, CXCL10, CCL5, CCL2
**CIRS**	IL-4, IL-10, sIL-1RA
**IRS+CIRS**	IL-1β, IL-6, TNF-α, CXCL8, CCL3, IL-2, IFN-γ, IL-12, IL-17, IL-15, G-CSF, GM-CSF, CXCL10, CCL5, CCL2, IL-4, IL-10, sIL-1RA
**Chemokines**	CXCL8, CCL11, CXCL10, CCL2, CCL3, CCL4, CCL5
**Growth factors**	FGF, PDGF, VEGF

IRS: immune-inflammatory response system; CIRS: compensatory immunoregulatory system
